# Repurposing the serotonin agonist Tegaserod as an anticancer agent in melanoma: molecular mechanisms and clinical implications

**DOI:** 10.1186/s13046-020-1539-7

**Published:** 2020-02-21

**Authors:** Wei Liu, Paweł Stachura, Haifeng C. Xu, Nikkitha Umesh Ganesh, Fiona Cox, Ruifeng Wang, Karl S. Lang, Jay Gopalakrishnan, Dieter Häussinger, Bernhard Homey, Philipp A. Lang, Aleksandra A. Pandyra

**Affiliations:** 1 Department of Molecular Medicine II, Medical Faculty, Heinrich-Heine-University, Universitätsstraße 1, 40225 Düsseldorf, Germany; 2 Institute of Immunology, Medical Faculty, University of Duisburg-Essen, Hufelandstrasse 55, 45147 Essen, Germany; 3 Institute of Human Genetics, Heinrich-Heine-University, Universitätsstrasse 1, 40225 Düsseldorf, Germany; 4 Department of Gastroenterology, Hepatology and Infectious Diseases, Heinrich-Heine-University, Moorenstrasse 5, 40225 Düsseldorf, Germany; 5 Department of Dermatology, Medical Faculty, Heinrich-Heine-University, Universitätsstraße 1, 40225 Düsseldorf, Germany

**Keywords:** Tegaserod, Melanoma, PI3K/Akt/mTOR pathway, Apoptosis

## Abstract

**Background:**

New therapies are urgently needed in melanoma particularly in late-stage patients not responsive to immunotherapies and kinase inhibitors.

**Methods:**

Drug screening, IC50 determinations as well as synergy assays were detected by the MTT assay. Apoptosis using Annexin V and 7AAD staining was assessed using flow cytometry. TUNEL staining was performed using immunocytochemistry. Changes in phosphorylation of key molecules in PI3K/Akt/mTOR and other relevant pathways were detected by western blot as well as immunocytochemistry. To assess in vivo anti-tumor activity of Tegaserod, syngeneic intravenous and subcutaneous melanoma xenografts were used. Immunocytochemical staining was performed to detect expression of active Caspase-3, cleaved Caspase 8 and p-S6 in tumors. Evaluation of immune infiltrates was carried out by flow cytometry.

**Results:**

Using a screen of 770 pharmacologically active and/or FDA approved drugs, we identified Tegaserod (Zelnorm, Zelmac) as a compound with novel anti-cancer activity which induced apoptosis in murine and human malignant melanoma cell lines. Tegaserod (TM) is a serotonin receptor 4 agonist (HTR4) used in the treatment of irritable bowel syndrome (IBS). TM’s anti-melanoma apoptosis-inducing effects were uncoupled from serotonin signaling and attributed to PI3K/Akt/mTOR signaling inhibition. Specifically, TM blunted S6 phosphorylation in both BRAF^V600E^ and BRAF wildtype (WT) melanoma cell lines. TM decreased tumor growth and metastases as well as increased survival in an in vivo syngeneic immune-competent model. In vivo, TM also caused tumor cell apoptosis, blunted PI3K/Akt/mTOR signaling and decreased S6 phosphorylation. Furthermore TM decreased the infiltration of immune suppressive regulatory CD4^+^CD25^+^ T cells and FOXP3 and ROR-γt positive CD4^+^ T cells. Importantly, TM synergized with Vemurafenib, the standard of care drug used in patients with late stage disease harboring the BRAF^V600E^ mutation and could be additively or synergistically combined with Cobimetinib in both BRAF^V600E^ and BRAF WT melanoma cell lines in inducing anti-cancer effects.

**Conclusion:**

Taken together, we have identified a drug with anti-melanoma activity in vitro and in vivo that has the potential to be combined with the standard of care agent Vemurafenib and Cobimetinib in both BRAF^V600E^ and BRAF WT melanoma.

## Background

Melanoma accounts for a large proportion of skin-related deaths and its incidence and mortality is on the rise [1, 2]. Despite advances in treatment options, the 5-year survival for patients suffering from late stage disease is only 20% [2]. The current therapeutic landscape encompasses surgery to remove early stage melanomas, traditional chemotherapy and radiation therapy for the more advanced stages, targeted therapies as well as immunotherapy. An increased understanding of the molecular landscape driving melanoma particularly activating mutations such as BRAF^V600E^ harbored by 50% of melanoma patients, has led to the development of small molecule inhibitors designed to specifically target multiple nodes of the MAPK pathway [3]. The approval of the Anti-CTLA checkpoint inhibitor Ipilimumab [4] in 2011 ushered immunotherapies focused on targeting the PD1/PD-L1 axis. This has had a tremendous impact on the therapy landscape in treating patients with advanced melanoma improving not only overall survival but leading to long-term survival in some patients. However, resistance to targeted therapies as well immunotherapy where bio-markers of response are not yet well-established [5, 6], present challenges in the treatment of melanoma. Although combinatorial approaches of the various targeted therapies together with immunotherapies are underway [7], the high-costs [5] associated with immunotherapy highlights an urgent need for novel anti-melanoma therapeutic options. The application of drugs used for alternate diseases as novel anti-cancer therapeutics, known as drug repositioning, has been successfully implemented in the clinical setting [8] and these compounds can be a rich potential source of novel, readily available anti-cancer therapeutics.

We conducted a pharmacologic screen composed of the NIH Clinical Collection (NCC) of 770 small molecules, FDA-approved or which have been previously used in human clinical trials to identify novel anti-melanoma agents. Each molecule was screened in the murine B16F10 cell line and its half maximal inhibitory concentrations (IC50) was determined. Amongst the compounds whose IC50 values were in the low micromolar range, Tegaserod (TM), a serotonin receptor 4 (HTR4) agonist, validated successfully in secondary screening approaches with BRAF WT and BRAF^V600E^ human melanoma cell lines and was pursued in further in vitro and in vivo studies. In melanoma, serotonin has been found to increase melanogenesis via HTR2A, an effect that was reversed by HTR antagonists [9]. And while HTR2B-C antagonists have been shown to inhibit migration in uveal [10] and metastatic melanoma [11], little is known about serotonin agonists, particularly HTR4 agonists in the context of this tumor type.

TM induced apoptosis in the B16F10 murine melanoma cell line as well as several human melanoma cell lines. In vivo*,* TM was well tolerated and efficacy was demonstrated in a syngeneic melanoma model testing primary tumor growth and metastasis. Importantly, TM strongly synergized with the standard of care BRAF^V600E^ targeting Vemurafenib in human melanoma cell lines harboring this mutation. Mechanistically, TM suppressed PI3K/Akt/mTOR signaling converging on the ribosomal protein S6 (S6) in vitro and in vivo. PI3K/Akt/mTOR inhibition was likely responsible for TM’s pro-apoptotic effects and anti-metastatic effects in melanoma cell lines as pharmacological inhibition of the pathway using specific inhibitors recapitulated the apoptotic phenotype confirming the sensitivity of melanoma cells to PI3K/Akt/mTOR pathway perturbation.

## Results

### A screen of pharmacologically active drugs identifies Tegaserod (TM) as having anti-melanoma activity

**Fig. 1:**
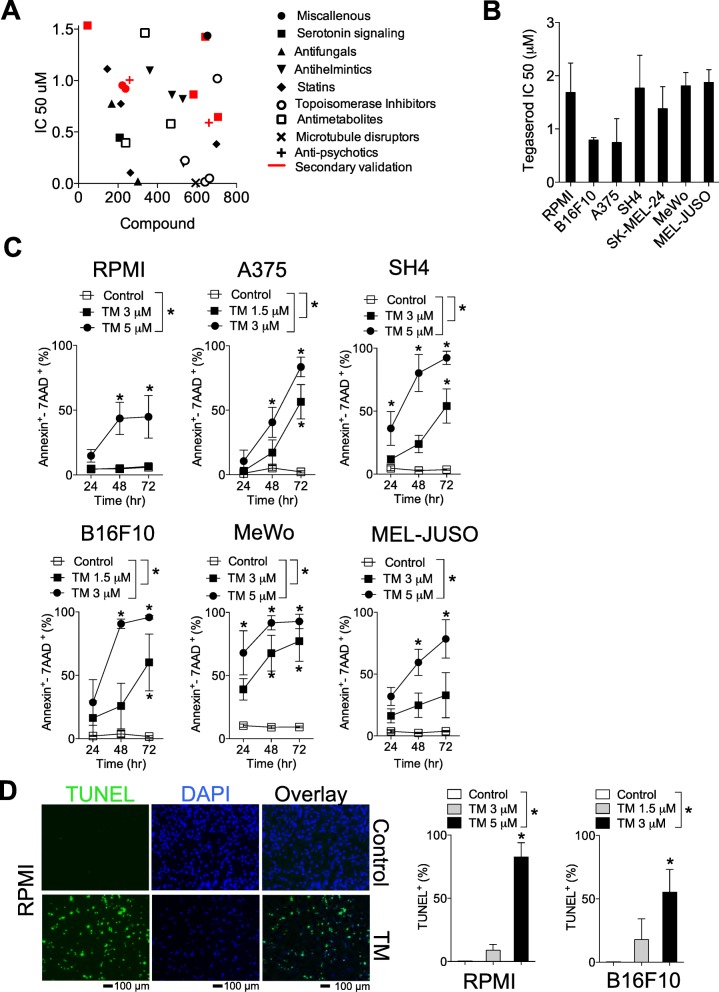
A pharmacological screen identifies Tegaserod (TM) as having anti-melanoma activity. **a** B16F10 murine melanoma cells were treated with 770 pharmacologically active compounds at a concentration range of 10 μM**-**78 nM. Several classes of compounds had anti-cancer activity with IC50 values in the low micromolar range as assessed by MTT assay following 72 h of exposure. **b** Tageserod (TM) a serotonin agonist was further validated and found to have anti-cancer effects in the B16F10 cell line and a panel of human malignant melanoma cell lines, A375, RPMI-7951 (RPMI), SH4, SK-MEL-24, MeWo and MEL-JUSO (n = 3–6). **c** Treatment with low micromolar doses of TM induced apoptosis in a time and dose-dependent manner as assessed by Annexin V/7AAD staining (n = 4–6). Percent apoptosis was ascertained by summing up the Annexin V^+^/7AAD^−^ and Annexin V^+^/7AAD^+^ populations. *P < 0.05 as determined by a 2-way ANOVA with a Dunnett’s post-hoc test. **d**, left panel Immunofluorescent TUNEL staining of RPMI cells 48 h post TM (5 μM) treatment is shown (A representative image of n = 3–5 is shown). *P < 0.05 as determined by a 1-way ANOVA with a Dunnett’s post-hoc test. Scale bar indicates 100 μm. **d**, right panel Quantification of the TUNEL apoptosis staining is shown (n = 3–5). Error bars in the all experiments indicate SEM

To identify drugs with novel anti-melanoma activities using an unbiased approach, we tested the NIH Clinical Collection (NCC) composed of 770 small molecules against the murine B16F10 (B16F10) melanoma cell line. A murine cell line was chosen with the intent of testing sensitivity in an in vivo immune-competent syngeneic model where immune cell-host interactions could also be evaluated. B16F10 cells were exposed to a concentration range (10 μM-78 nM) for 72 h and the IC50 values for each compound were determined by assessing cell viability at each dose using the MTT assay (Additional file 1: Figure S1A). From the compounds with determinable IC50 values, many had IC50 values in the low micromolar range (< 2 μM) that could be subdivided into broad pharmacological and/or functional classes (Fig. 1a). Positive hits included members of the statin, antifungal and antihelmintics categories, most of which are already being pre-clinically evaluated as therapeutics in melanoma or other cancers [12–14]. Others, belonging to the microtubule disruptors, antimetabolite and topoisomerase inhibitors are already in use as anti-cancer agents [15]. Secondary screening validation focused on compounds in the serotonin signaling categories. Tegaserod (TM), a serotonin agonist had IC50 values in the low micromolar ranges in B16F10 cells as well as several human malignant melanoma cell lines (Fig. 1b). The chosen melanoma cell lines have both wildtype (WT) and mutated BRAF. Specifically, the A375, SH4, RPMI-7951 (RPMI) and SK-MEL-24 harbor the BRAF^V600E^ mutation while the B16F10 murine cells and the human MeWo and MEL-JUSO cell lines are BRAF WT. As the MTT assay is only an indirect indicator of cell viability, we next assessed whether TM is capable of inducing apoptosis. There was a significant time and dose-dependent increase in apoptosis in all cell lines tested as determined by measuring Annexin V and 7AAD staining following treatment with TM (Fig. 1c).

To further verify and characterize cell death observed following treatment of melanoma cells with TM, we assessed apoptosis using TUNEL staining in representative BRAF^V600E^ and BRAF WT melanoma cell lines, RPMI and B16F10 cell lines respectively. Treatment with TM induced an increase in TUNEL staining relative to untreated controls (Fig. 1d). Taken together, we have identified a compound with previously unknown anti-melanoma activity that induces apoptosis in melanoma cell lines.

### Tegaserod (TM) exerts its anti-cancer effects independently of serotonin signaling

**Fig. 2:**
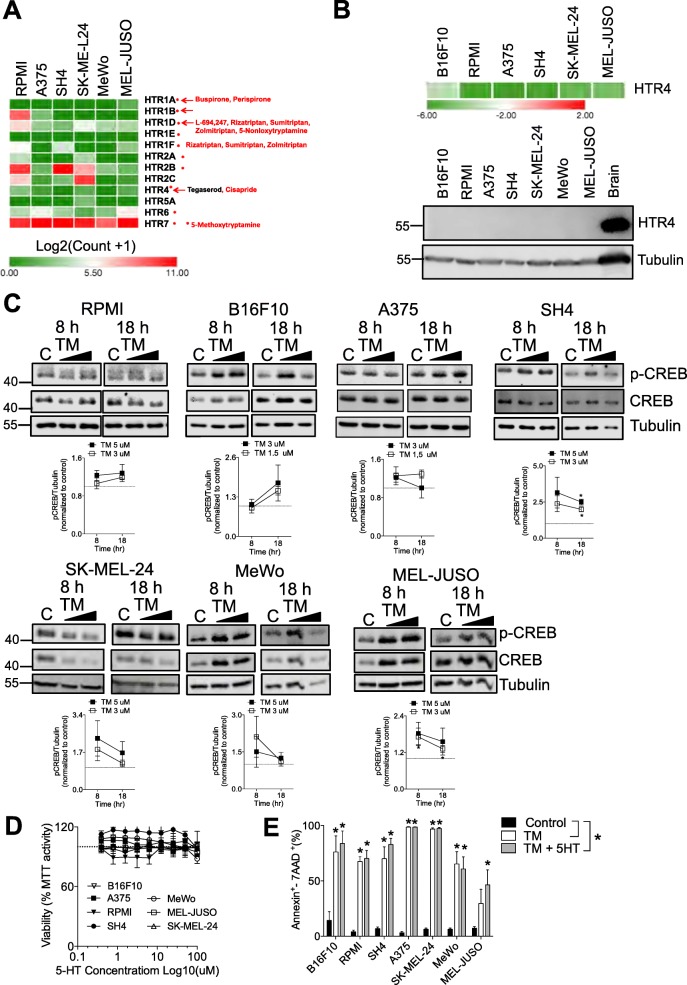
TM induces apoptosis independently of serotonin signaling (**a**) Expression of the different serotonin receptors (5-HTRs) in our panel of human melanoma cell lines. Data was mined from the Cancer Cell Line Encyclopedia. **b**, upper panel mRNA expression of 5-HTR4 which is targeted by TM is shown. Expression values are represented as Log10 (CT*HTR4*- CT*GAPDH*) and visualized through Morpheus software (Broad Institute) (n = 3–5). **b**, lower panel Protein expression of HTR4 in melanoma cell lines is shown using mouse brain as a positive control (A representative immunoblot of n = 3 is shown). **c**, upper panel Changes in phosphorylation of the transcription factor CREB 8 and 18 h post TM treatment are shown (A representative immunoblot of n = 3–5 is shown). Quantification of immunoblots is shown in C (lower panel). **d** Treatment with serotonin (5-HT) for 72 h did not have anti-proliferative effects in melanoma cells (n = 3–4). **e** Co-treatment of TM (3 μM for B16F10 and A375 and 5 μM for RPMI, SH4, MeWo and MelJuso melanoma cells) with serotonin (5-HT, 100 μM) did not impact the anti-melanoma effects of TM and did not alter TM induced apoptosis as assessed 72 h post treatment using the Annexin V/7AAD assay (*n* = 3–6). Error bars in the all experiments indicate SEM; *P < 0.05 as determined by a Student’s t-test (unpaired, 2 tailed), or a one-way ANOVA with a Dunnett’s post-hoc test

We wondered whether melanoma cancer cell lines express serotonin receptors 5-HTRs. We mined expression data from the Cancer Cell Line Encyclopedia (CCLE) [16] and found that some receptors particularly HTR7 have a high expression relative to the others in the human melanoma cell lines used in our system (Fig. 2a). TM was synthesized with the primary intent of functioning as a 5-HTR4 agonist [17]. HTR4 mRNA was weakly detected (not detectable in the MeWo cell line) but HTR4 protein expression was undetectable in all melanoma cell lines tested (Fig. 2b).

The main transduction mechanisms of the G-coupled 5-HTR1 and 5-HTR4–7’s occur through modulation of cAMP levels [18]. We therefore wondered whether TM alters cAMP levels in melanoma cell lines. Treatment of melanoma cell lines with TM did not alter cAMP levels (Additional file 1: Figure S2A). The expression of genes that have been previously shown to be upregulated upon serotonin (5-HT) treatment through PKA signaling, *PDE2A*, *MET*, *TREM1*, *THBS1*, *SERPINB2* and *S1PR1* [19] was not changed following TM treatment (Additional file 1: Figure S2B). As expected, with the lack of change in cAMP levels, there was no significant increase in the phosphorylation of the cAMP response element binding protein (CREB) in RPMI, B16F10, A375, SK-MEL-24, MeWo cells although p-CREB was increased in SH4 and MEL-JUSO cells (Fig. 2c). To further address the question of whether serotonin agonist signaling is responsible for the apoptotic phenotype, we treated melanoma cancer cells with a wide range (100 μM–0.4 μM) of 5-HT. Treatment with 5-HT had little effect on the melanoma cells (Fig. 2d) and co-treatment of 5-HT with TM had no effect on apoptosis induced by TM (Fig. 2e). Taken together, the anti-melanoma effects caused by TM are likely not being mediated through 5-HTR4 signaling.

### Tegaserod (TM) blunts of ribosomal protein S6 (S6) phosphorylation through the PI3K/Akt/mTOR pathway

We wondered what signaling pathways perturbed by treatment with TM are responsible for the apoptotic phenotype in melanoma cells.

**Fig. 3:**
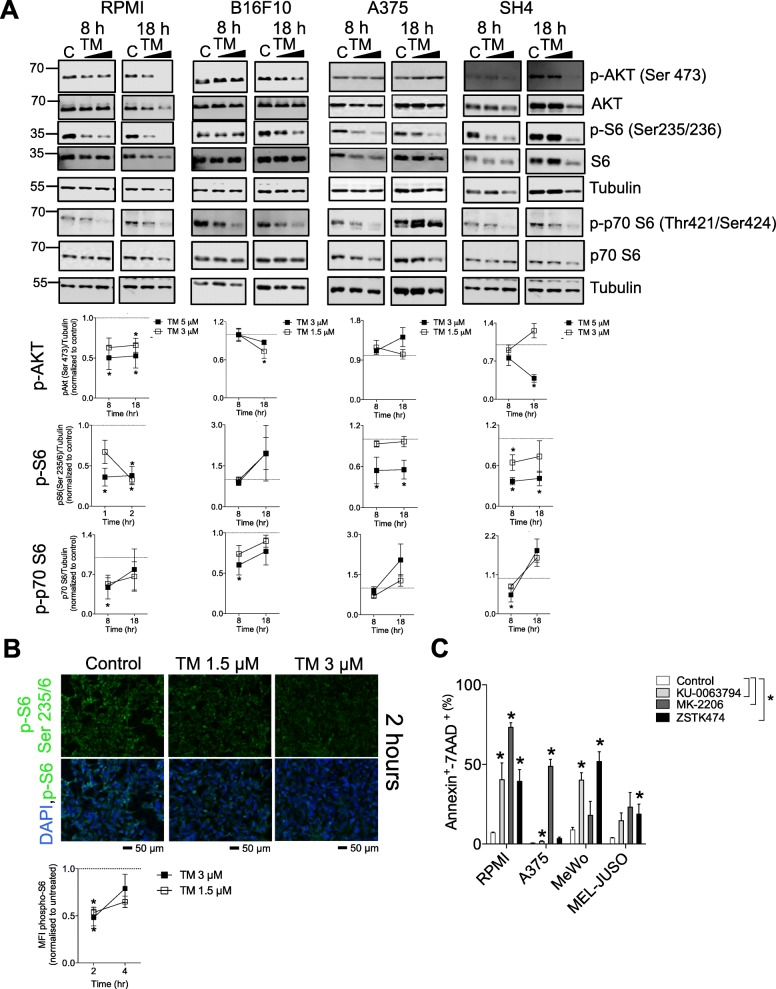
TM blunts ribosomal protein S6 (S6) phosphorylation through the PI3K/Akt/mTOR pathway. **a** Treatment with increasing doses of TM at the indicated time-points prior to apoptosis induction decreased phosphorylation of Akt (p-Akt) at Ser 473, phospho-S6 (p-S6) at Ser235/6 and phospho-p70 S6 (p-p70 S6) in RPMI, B16F10, A375 and RPMI cells (representative immunoblots of n = 3–7 are shown) and quantified below. **b** Immunofluorescent p-S6 staining of B16F10 cells treated with TM for 2 h is shown (A representative image of n = 3 is shown) and quantified in B, lower panel). Scale bar indicates 50 μm. **c** Treatment for 48 h with the PI3K inhibitor ZSTK474 (2 μM for MeWo, 6 μM for MEL-JUSO and A375 and 1 μM for RPMI), pan-Akt inhibitor MK-2206 (2 μM for MeWo, 6 μM for MEL-JUSO, 10 μM for A375 and 4 μM for RPMI) and mTORC1/mTORC2 inhibitor KU-0063794 (2 μM for MeWo and 4 μM for all other cell lines) induced apoptosis in melanoma cells as assessed by Annexin V/7AAD staining (n = 3–6). Percent apoptosis was ascertained by summing up the Annexin V^+^/7AAD^−^ and Annexin V^+^/7AAD^+^. Error bars in the all experiments indicate SEM. *P < 0.05 as determined by a Student’s t-test (unpaired, 2 tailed) or a one-way ANOVA with a Dunnett’s post-hoc test

Common driver oncogenic pathways critical to melanoma pathogenesis are the MAPK and PI3K/Akt and mTOR pathways [20]. ERK phosphorylation was not significantly affected following treatment of melanoma cells with TM at early time-points, 8 and 18 h, prior to apoptosis induction (Additional file 1: Figure S3). Phosphorylation of ribosomal protein S6 (S6) on the Ser^235/236^ phosphorylation sites was inhibited in all human melanoma cell lines tested (Fig. 3a and Additional file 1: Figure S4A). Phosphorylation of S6 on the Ser^240/244^ phosphorylation sites was also inhibited in the RPMI and SH4 cells lines (Additional file 1: Figure S4B). As there was no difference in S6 phosphorylation between control and TM treated B16F10 cells at 8 and 18 h we also assessed earlier time-points. At 2 and 4 h post TM treatment, p-S6 was also blunted as assessed by immunofluorescence staining (Fig. 3b).

S6 is phosphorylated by the p70 S6 kinase directly downstream of the mammalian target of rapamycin (mTOR) complex 1 (TORC1) [21]. TORC1 converges on multiple upstream signaling pathways including the MAPK [22] and PI3K/Akt /mTOR pathways [23–25]. The MAPK pathway activity, as assessed by ERK phosphorylation was unperturbed in response to TM treatment (Additional file 1: Figure S3). Through the PI3K/Akt pathways, activated Akt can activate TORC1 through tuberous sclerosis complex 2 (TSC2) or PRAS40 phosphorylation [25, 26]. AKT phosphorylation on Ser473 was suppressed at 8 or 18 h post treatment with TM in RPMI, SH4 and B16F10 cells (Fig. 3a). Not surprisingly, phosphorylation of the kinase directly upstream of S6, p70 S6 at Thr^421^/Ser^424^, was also decreased in RPMI, B16F10 and SH4 cells post TM treatment (Fig. 3a). Maximal Akt activation occurs through phosphorylation of two key residues, Ser 473 by mTORC2 [27] or DNA-dependent protein kinase (DNA-PK) [28] and by phosphoinositide-dependent kinase 1 (PDK1) at Thr^308^ [29]. However, as PDK1 phosphorylation at Ser^241^ was not blunted following treatment with TM (Additional file 1: Figure S4B) and phospho-Akt at residue Thr 308 was not detectable in our system under normal cell growth conditions (data not shown) Akt activity by TM might be rather suppressed through mTORC2 or DNA-PK. However, there is the possibility that suppression of phosphorylation at alternative Akt sites occurs through other regulators such CK2 [30] or GSK-3α [31] and this would have to be further explored.

To confirm that melanoma cells used in our system are sensitive to PI3K/Akt/mTOR inhibition, we treated melanoma cells with specific inhibitors of AKT (MK-2206, a highly selective Akt1/2/3 inhibitor), PI3K (ZSTK474, a class I PI3K isoforms inhibitor) and mTOR (KU-0063794, a specific dual-mTOR inhibitor of mTORC1 and mTORC2). All our tested melanoma cell lines both BRAF^V600E^ and BRAF WT were sensitive to AKT, PI3K and pan-mTOR inhibition with IC50 values in a similar range as that of TM (Additional file 1: Figure S5 and Table 1). ZSTK474 and/or MK-2206 and/or KU-0063794 also induced apoptosis in both BRAF^V600E^ and BRAF WT melanoma cell lines (Fig. 3c). Taken together, TM suppresses p-S6 through blunting PI3K/Akt/mTOR signaling in melanoma cells, an effect that is likely responsible for the pro-apoptotic effects observed as treatment with various inhibitors of the pathway was able to recapitulate the phenotype.

**Table 1:** Melanoma cell line sensitivity to PI3K/Akt and mTOR pathway inhibition

Compound	Target	IC50±SEM					
		B16.F10	A375	RPMI	SH4	MeWo	MEL-JUSO
MK-2206	Pan-AKT	0.29±0.05	4.76±0.58	1.92±0.36	3.11±0.91	1.26±0.01	3.03±0.35
ZSTK474	PI3K	0.95±0.33	2.69±1.14	0.51±0.05	2.80±0.81	1.06±0.07	3.47±0.28
KU-0063794	Pan-mTOR	0.68±0.26	1.90±1.45	1.63±0.28	1.71±0.86	0.97±0.05	1.84±0.12

### Tegaserod (TM) delays tumor growth, reduces metastases, increases survival and suppresses p-S6 in vivo

**Fig. 4:**
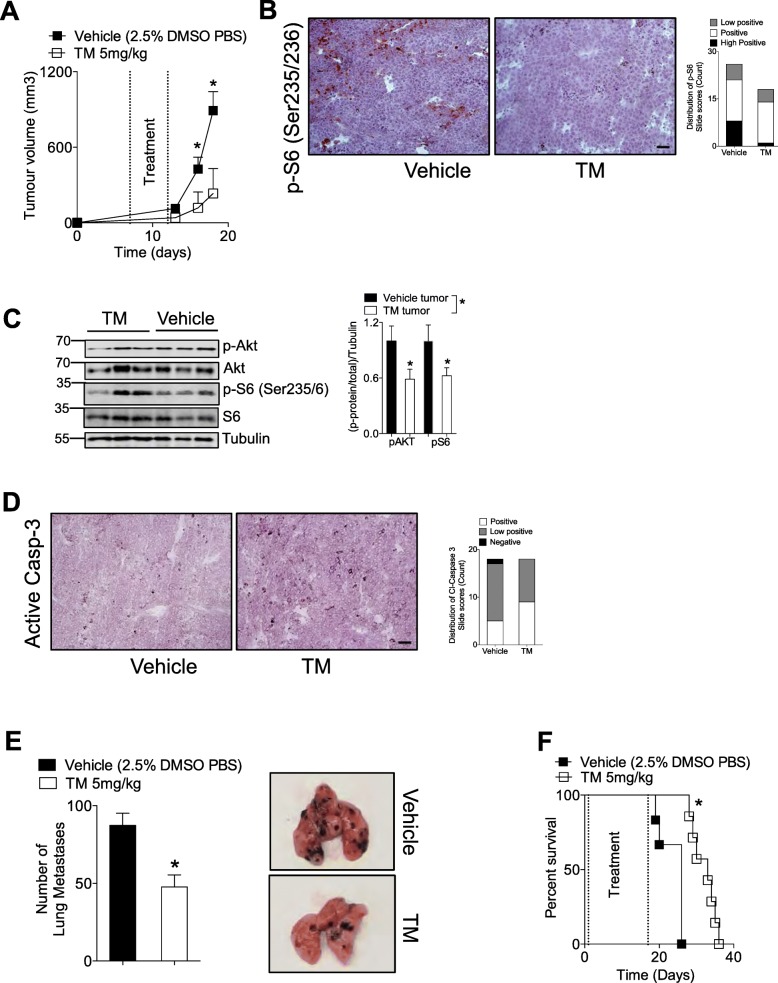
Tegaserod (TM) delays tumor growth, induces tumor cell apoptosis and inhibits Akt and p-S6 phosphorylation in vivo. **a** C57BL/6 J mice were subcutaneously injected with 5 × 10^5^ B16F10 cells. Seven days post-tumor injection, mice were randomized and into two groups and treated daily with 5 mg/kg of Tegaserod or vehicle for five consecutive days. **a** Tumor volume was measured for 18 days after which mice were sacrificed (n = 6–8). **b** Mice were sacrificed on Day 13 post tumor-inoculation and immunohistochemical staining of tumor tissue for p-S6 is shown (a representative image of n = 6 mice is shown). A third of pictures from tumors of mice treated with vehicle were classified as ‘high positive’ for p-S6 compared to only 1 slide from TM treated mice (3–5 pictures from different fields of view were obtained of tumors from each independent mouse, for a total of 26 and 18 tumor pictures for vehicle and TM treated mice respectively). **c**, left panel Immunoblots of tumor lysates from TM or control treated mice confirmed decreased Akt (Ser473) and S6 (Ser235/6) phosphorylation (n = 6–9 mice, with 3 mice being shown on one immunoblot) quantified in **c**, right panel. **d** Mice were sacrificed on Day 13 post tumor-inoculation and **d**, left panel immunohistochemical staining of tumor tissue revealed that tumors of mice treated with TM had increased active Caspase-3 expression (a representative image of n = 6 is shown). **d**, right panel. The relative score distribution of tumor slides is shown (3 pictures from different fields of view were obtained of tumors from each independent mouse (n = 6), for a total of 18 tumor pictures for each stain and treatment group. **e** C57BL/6 J mice were intravenously injected with 2 × 10^5^ B16F10 cells. Starting at day 1 post inoculation, mice were treated with 5 mg/kg of Tegaserod or vehicle three times a week. Mice were sacrificed at Day 14 post tumor inoculation and lung metastases counted with representative lung images shown in the right panel (n = 10). **f** C57BL/6 J mice were intravenously injected with 10^5^ B16F10 cells. Starting at day 1 post inoculation, mice were treated with 5 mg/kg of Tegaserod or vehicle three times a week till day 17 post-inoculation. Mice were monitored for survival (n = 6-7). All Scale bars indicate 50 μm. Error bars in the all experiments indicate SEM. *P < 0.05 as determined by a determined by a Student’s t-test (unpaired, 2 tailed) or and log-rank test for analysis of Kaplan Meier survival curves

To evaluate the efficacy of TM against melanoma tumor growth we used a syngeneic immune-competent model. Mice were subcutaneously inoculated with B16F10 cells, and 7 days later, randomized and treated with daily injections of TM or vehicle for 5 days. Treatment significantly decreased tumor growth (Fig. 4a) and resulted in only slight decreases in weight following treatment (Additional file 1: Figure S6A). There were no changes in liver damage markers AST, LDH and ALT (Additional file 1: Figure S6B). The in vitro TM-mediated PI3K/Akt/mTOR signaling inhibition was re-capitulated in vivo*.* When immunohistochemical staining of tumor tissue harvested 13 days post inoculation was performed for phosphorylation of S6 (Ser235/236), one third of control tumor slides were classified as having a high positive score. This is sharp contrast to tumors from TM treated mice where only one slide scored as having a high positive score (Fig. 4b). Images were scored for positive staining using the IHC profiler which employs an automated, unbiased approach to evaluate antibody staining in tissue sections [32]. Furthermore, tumor lysates from TM treated mice had significantly lower Akt and S6 phosphorylation levels (Fig. 4c).

To assess tumor apoptosis, immunohistochemical staining of tumor tissue harvested 13 days post inoculation was performed for active Caspase-3 and cleaved Caspase-8 (Fig. 4d and Additional file 1: Figure S6D). 50% of tumor slides from TM-treated mice stained for active Caspase-3 had a positive score and the other 50% were scored as low positive. In contrast, tumor slides from vehicle-treated mice were 5% negative for cleaved Caspase-3, and only 27% scored positive and 68% scored low positive (Fig. 4d). There was a significantly higher contribution from the high positive stained areas for active Caspase-3 in the tumors of TM-treated mice (Additional file 1: Figure S6C) indicating that TM treatment caused tumor cell apoptosis in vivo. When tumor lysates were probed for cleaved Caspase-8, tumors from TM treated mice demonstrated a trend towards increased cleaved Caspase-8 but differences were not significant (Additional file 1: Figure 6E).

To evaluate the ability of TM to decrease metastasis in vivo, we intravenously injected B16F10 melanoma cells into C57BL/6 J mice and monitored lung metastases in control and TM treated mice. Mice treated with TM had significantly less lung metastases (Fig. 4e). As a result, the mice treated with TM survived significantly longer than control mice (Fig. 4f). Taken together, we have shown that in vivo TM is well tolerated, can retard tumor growth, induces tumor apoptosis and blunts p-S6.

### Tegaserod (TM) decreases the infiltration of regulatory T cells and synergizes with BRAF and MEK inhibitors

**Fig. 5:**
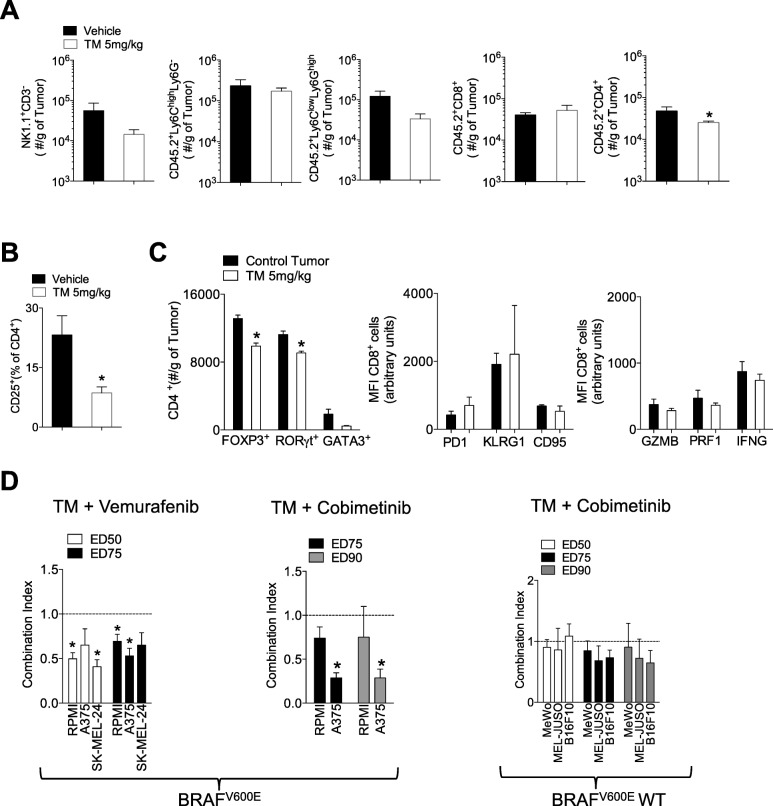
Tegaserod (TM) decreases tumor infiltration of CD25^+^CD4^+^ T cells and synergizes with Vemurafenib and Cobimetinib. **a-c** C57BL/6 J mice were subcutaneously injected with 5 × 10^5^ B16F10 cells. Seven days post-tumor injection, mice were randomized and into two groups and treated daily with 5 mg/kg of Tegaserod or vehicle for five consecutive days. Mice were sacrificed on Day 13 post tumor-inoculation and tumor infiltrating lymphocytes using were assessed using FACS analysis (n = 3–6). **d** Melanoma cell lines harboring the BRAF^V600E^ mutation, A375, RPMI-7951 (RPMI) and SK-MEL-24 were exposed to a dose range of TM and Vemurafenib in a fixed 1:1 ratio. BRAF^V600E^ and BRAF WT melanoma cell lines were exposed to a dose range of TM and Cobimetinib in a fixed ratio (RPMI, 1:2, A375 64:1, MeWo 4:1, MEL-JUSO 4:1, B16F10 1:1). Synergy was evaluated using the combination index (CI) from the dose-response curves. CI < 1 indicates synergy, CI = 1 indicates additivity, and CI > 1 indicates antagonism. The EC50 (50% effective concentration) and EC75 (75% effective concentration) or EC90 (90% effective concentration) are shown (n = 3–6). *P < 0.05 as determined by a one-sample Student’s t-test

Next, we wondered whether TM treatment impacted immune infiltrates. We harvested tumors from mice at day 13 post inoculation when there were no significant differences in tumor size and found that the numbers of NK1.1^+^CD3^−^ natural killer (NK) cells, Ly6C^high^Ly6G^−^ monocytes, Ly6C^low^Ly6G^high^ granulocytes and CD8^+^ T cells were not different between tumors harvested from control and TM treated mice (Fig. 5a). However, tumors harvested from TM treated mice were characterized by lower amounts of infiltrating CD4^+^ T cells (Fig. 5a). As regulatory CD4^+^CD25^+^ T cells play a crucial role in suppressing anti-tumoral immunity [33] and have been shown to be susceptible to PI3K/PTEN/mTOR axis inhibition [34], we next checked whether there were any differences in the percentage of infiltrating regulatory CD4^+^CD25^+^ T cells between TM treated and control tumors. Not only was the percentage of CD4^+^CD25^+^ T cells lower in tumors harvested from TM-treated mice (Fig. 5b), but the infiltration of FOXP3 expressing CD4^+^ cells was decreased (Fig. 5c). By contrast, surface markers of exhaustion (PD-1), activation (KLRG1, Granzyme B, perforin, Interferon gamma (IFNγ)) and death (CD95) were no different on tumor infiltrating CD8^+^ T cells between TM and vehicle treated mice (Fig. 5c).

Any potential novel therapy will not be used as in a mono-therapeutic setting but will be combined with the current standard of care. We therefore ascertained whether TM could be combined with Vemurafenib, a B-Raf inhibitor approved for the treatment of late-stage melanoma [35]. We tested the combination in human cell lines harboring the BRAF^V600E^ mutation targeted by Vemurafenib, namely RPMI, A375 and the SK-MEL-24 cells. TM synergized with Vemurafenib in all cell lines tested (Fig. 5d). Other kinase inhibitors currently in use for the treatment of late stage melanoma include the MEK inhibitor Cobimetinib. TM also synergized with Cobimetinib in A375 cells at higher effective doses (ED75 and ED90) and was additive in RPMI, B16F10, MeWo and MEL-JUSO melanoma cell lines (Fig. 5d). Taken together, we have shown that TM inhibited tumor growth in vivo and can be successfully combined with the current standard of care.

## Discussion

Our screen identified several potential hits with anti-melanoma activity including serotonin agonists and other compounds, such as statins, antihelmintics and antifungals which are already being re-purposed as anti-cancer agents pre-clinically or in the clinical setting. The serotonin signaling class of compounds that were positive hits in the original screen included serotonin agonists as well as the anti-depressants indatraline and maprotiline. The latter two are multi-functional and not only prevent the re-uptake of serotonin but also dopamine and norepinephrine and did not have appreciable anti-melanoma activity when compared to the other compounds in the serotonin signaling class including TM. Serotonin signaling occurs when serotonin, a neurotransmitter present in the gut, blood platelets and the central nervous system (CNS), binds to serotonin receptors (5-HTRs) resulting in complex physiological and behavioral changes affecting mood, cognition, digestion, pain perception [18, 36]. The pharmacological opportunities to modulate these physiological processes and impact human disease are vast and have resulted in a plethora of 5-HTR agonist and antagonist ligands. There are seven families of human serotonin receptors mostly part of the G-protein coupled receptor family differentially expressed throughout the CNS, liver, kidney, heart, gut [18]. We were intrigued by the possibility of investigating TM because the role of serotonin signaling in cancer remains controversial. Serotonin and 5-HTR2A agonists were found to induce melanogenesis in melanoma cell lines [9]. Jiang et al. reported increased levels of serotonin and 5-HTR2B in human pancreatic ductal adenocarcinomas which promoted pancreatic tumor growth in mice [37]. Many other studies have similarly reported growth stimulatory effects of serotonin signaling through various 5-HTRs and inhibitory effects of 5-HTR antagonists in many tumor types [38, 39]. However, there have also been reports, albeit much fewer, suggesting that treatment with serotonin agonists might also have anti-cancer effects in glioma [40] and breast cancer cells [41]. Involvement within the autocrine loops and activation of the MAPK, JNK, PI3K/Akt/mTOR [37, 38] pathways has been implicated in serotonin’s mitogenic role.

We did not observe any pro or anti-mitogenic effects following treatment with serotonin (5-HT) in melanoma cells. Co-treatment of TM with 5-HT did not effect the compound’s ability to induce apoptosis in the melanoma cells. This suggests that the affinity of the synthetic ligand TM is stronger for the 5-HTR’s than for the natural ligand 5-HT, and/or that the pro-apoptotic effects of TM can be uncoupled from serotonin signaling. Treatment with 5-HTR ligands, agonists or antagonist presents a complex scenario. As previously reported [38] treatment with one ligand can yield opposing concentration dependent results. Serotonin signaling following TM treatment might occurring through other 5-HTRs. TM has been reported to be an agonist for the 5-HTR1A-D and an antagonist for 5-HTR2A-B [42]. In our case, we used doses in the low micromolar range, high enough to elicit tumor apoptosis inducing pleiotropic effects [42, 43]. Although we did not observe significant changes in cAMP levels and 5-HT responsive genes following TM treatment in most of the cell lines, increased p-CREB levels were observed in the SH4 and MEL-JUSO cells suggesting a possible involvement of other serotonin receptors including ones previously unidentified as being targets for TM. However, other antagonists and agonists present in the screen including the 5-HTR4 agonists (Cisparide) that did not have any anti-cancer effects further suggesting that TM is uniquely acting to distinctly target other molecules, likely upstream receptors or kinases of the PI3K/Akt/mTOR pathways.

The current repertoire of clinically approved treatment options in melanoma encompasses agents that inhibit proliferation and induce cell death [44]. This includes targeted inhibitors of the BRAF pathway and checkpoint inhibitors. The former class of agents such as Vemurafenib cause cell arrest and trigger apoptosis [35, 45] while the checkpoint inhibitors cause immunogenic cell death through lytic and apoptotic cell death mediated by activated CD8^+^ T and NK cells respectively [6, 44]. Resistance to the targeted inhibitors and variable checkpoint inhibitor response rates has shifted the focus in recent years interest to finding novel combination treatments to overcome resistance and increase response rates [7]. Strategies include targeting other forms of cell death such as necroptosis [46], inhibiting MAPK reactivation that occurs following targeted therapy treatment, and concomitantly inhibiting other pathways including the PI3K/Akt/mTOR [47, 48].

Recently, a report has shown phosphorylation of S6 to be marker of sensitivity to BRAF mutated melanoma and that suppression of S6 after MAPK treatment was a predictor of progression-free survival [23]. In our investigation, TM’s suppression of p-S6 and its strong synergy with vemurafenib in BRAF mutated human melanoma cell lines is in accordance with the above report. Importantly, TM also suppressed S6 phosphorylation in non-BRAF mutated melanoma cell lines indicating a broader therapeutic potential of TM in patients without the BRAF mutation but where the PI3K/Akt/mTOR pathway is activated such as in patients harboring NRAS mutations [48]. The suppression of S6 phosphorylation is likely mediated by decreased mTORC1 activity as phosphorylation of the direct upstream regulator of S6, p70 S6 kinase was also blunted. mTORC1 integrates several upstream pathways related to cellular growth and metabolism including MAPK through RSK [22], PI3K/Akt [25] as well as the liver kinase B1 (LKB1)-adenosine monophosphate-activated protein kinase (AMPK) [49]. As TM did not perturb the MAPK pathway but decreased Akt phosphorylation at a residue known to be phosphorylated by mTORC2 [27], it’s likely that S6 is affected through the PI3K/Akt pathway although the potential contribution of AMPK would also have to be explored. Interestingly, Yoon et al. found that dual mTORC1/2 inhibition following treatment with Torin1 in A375 melanoma cells induced focal adhesion re-organization, increased the size of focal adhesions and increased migration and invasion *in vitro* [29]. TM did not phenocopy Torin 1 using B16F10 cells, as treatment with TM decreased the number metastases in vivo in an immuno-competent murine model where the presence of tumor infiltrating lymphocytes was considered. The immunosuppressive and pro-tumorigenic contribution of regulatory CD4^+^ T cells in the tumor microenvironment is well established [50]. As the infiltration of FOXP3 expressing CD4^+^ T cells and regulatory CD4^+^ T cells in TM treated tumors was decreased, this likely contributes to TM’s anti-cancer effects in vivo*.*

Tegaserod (Zelnorm, Zelmac) which is used for the treatment of irritable bowel syndrome (IBS) [51, 52] was also shown to be effective against chronic constipation [53]. Although Tegaserod was well-tolerated and effective, it was removed off the market in the Unites States in 2007 at the FDA’s request [54] chiefly due cardiovascular (CV) safety concerns raised through retrospective clinical trial analysis. However, all adverse cardiovascular events occurred in patients with CV disease and/or CV risk factors. Furthermore, the link between Tegaserod and adverse CV outcomes was not recapitulated in subsequent epidemiological studies [55, 56] which found no association between Tegaserod use and adverse CV’s. The tolerability and availability of the drug would likely outweigh the relatively low cardiovascular risk (0.1%) associated with Tegaserod usage especially in melanoma patients with few treatment options. In vivo*,* TM retarded decreased metastatic and primary tumor growth, induced apoptosis and suppressed p-Akt and p-S6 in tumor cells. TM is available in generic form and has the potential to be re-purposed as an anti-melanoma agent. The dose we used in mice, 5 mg/kg, once daily, is roughly equivalent to a Human Equivalent Dose (HED) [57] of 0.405 mg/kg. Given that TM is available as a 6 mg pill administered twice daily, the doses we used in our in vivo studies are within the physiological range. Furthermore, as the compound synergized with Vemurafenib and other kinase inhibitors currently used in melanoma patients with late-stage disease, this is likely a favorable point of clinical entry especially since most patients eventually develop resistance to Vemurafenib and other kinase inhibitors [7, 47]. Furthermore, as the BRAF WT cohort of patients are a diverse group, treatment options are much less clear cut [44, 58] although immunotherapies, as with BRAF^V600E^ melanoma are a promising albeit costly treatment approach [59]. Currently there are a lot of different combinations in clinical trials using MEK in combination with inhibitors of the PI3K/AKT/mTOR axis [58] (NCT01941927, NCT01363232, NCT01337765).

## Conclusions

Taken together, we have identified a compound that is effective in inducing apoptosis in both BRAF^V600E^ and BRAF WT melanoma and has the potential to be readily translated to the clinic especially in the case of BRAF WT melanoma where fewer approved treatment options exist. Tegaserod blunted phosphorylation of S6 through inhibition of the PI3K/Akt/mTOR pathway in vitro and in vivo*.* Tegaserod synergized with Vemurafenib in BRAF^V600E^ human cell lines and could also be combined with Cobimetinib in BRAF WT cell lines.

## Materials and methods

### Cell culture and compounds

B16F10, A375, SH4, RPMI-7951 and SK-MEL-24 melanoma cell lines were purchased from ATCC. MeWo and MEL-JUSO cell lines were kindly provided by Dr. A. Roesh (Universitätsklinikum Essen, Essen, Germany). The MEL-JUSO and MeWo cell lines were both originally purchased from ATCC. B16F10 murine cells, A375 and SH4 human malignant melanoma cell lines were maintained Dulbecco Modified Eagle’s Medium (DMEM). Human RPMI-7951 malignant melanoma cells were maintained in Eagle’s MEM. SK-MEL-24 were maintained in Eagle in Earle’s BSS with non-essential amino acids. MeWo and MEL-JUSO cell lines were maintained in Roswell Park Memorial Institute (RPMI) medium. All media were supplemented with 10% FCS (15% for SK-MEL-24) and penicillin streptomycin. Cells were incubated at 37 °C in 5% CO^2^, and all cell lines were routinely confirmed to be mycoplasma-free (MycoAlert Mycoplasma Detection Kit, Lonza). The NIH Clinical Collection (NCC) composed of 770 small molecules mainly dissolved in DMSO at a concentration of 10 μM was obtained from the NIH, Tegaserod (Sigma) was dissolved in DMSO, serotonin (Sigma) was dissolved in water. MK-2206, ZSTK474, KU-0063794, Vemurafenib, Cobimetinib (Selleckchem) were dissolved in DMSO.

### MTT assays

For the MTT colorimetric assay, cells were seeded in 96 well plates and viability was assessed following addition of the MTT (Sigma) reagent. Half-maximal inhibitory concentrations (IC50) values were computed from dose–response curves using Prism (v5.0, GraphPad Software).

### Flow Cytometry

For Annexin V/7AAD apoptosis assays, trypsinized cells were washed and stained in Annexin V binding buffer (BD Biosciences). Melanoma cells were treated at doses of 2 x – 4x IC50 values for TM and 2 x IC50 for PI3K/Akt/mTOR inhibitors. Stainings of CD4^+^ cells for FOXP3, RORγ and GATA3 and of CD8^+^ cells for Granzyme B, perforin and IFNγ were performed using the Foxp3 mouse Treg cell staining buffer kit (eBioscience). Cells were analyzed using FACS (FACS Fortessa, BD Biosciences).

### Immunofluorescence

For TUNEL staining, cells were seeded on cover slips, treated and 48 h later fixed by 4% formaldehyde in PBS for 30 min, permeabilized with 0.1% Triton X-100, 0.1% sodium citrate in PBS for 2 min and stained using the TUNEL staining kit as per manufacturer’s protocol (Roche). For p-S6 staining, cells seeded on cover slips were stained with primary anti-p-S6 antibody (Ser 235/6, Cell Signaling) overnight, followed by incubation with secondary anti-Rabbit IgG Cy3 conjugate antibody. Cover slips were incubated with DAPI in PBS for 30 min. Images were taken with an Axiocam 503 color microscope (ZEISS).

### Immunoblotting

Cells were lysed using boiling hot SDS lysis buffer (1.1% SDS, 11% glycerol, 0.1 mol/L Tris, pH 6.8) with 10% β-mercaptoethanol. Tumor tissue was crushed using a tissue lyser (TissueLyser II, QIAGEN) and cells were gently lysed using Triton X-100. Blots were probed with anti-α-tubulin (Merck), anti-HTR4 (ThermoFischer), anti-cleaved Caspase 8, anti-Akt, anti-p-Akt (Ser 473), anti-S6, anti-p-S6 (Ser235/6, Ser240/4), anti-p70 S6, anti-p-p70 S6 (Thr421/Ser424), anti-p-ERK1/2, anti-ERK1/2, anti-p-CREB (Ser133) and anti-CREB (all from Cell Signaling) and detected using the Odyssey infrared imaging system (Odyssey Fc, LI-COR Biosciences). Immunoblots were quantified using ImageJ.

### Combination index (CI) determination

Synergy between TM and Vemurafenib, and Cobimetinib was evaluated by calculating the CI [60]. Dose–response curves were generated for TM, Vemurafenib and Cobimetinib alone and each drug in combination with TM at a constant ratio following compound exposure for 72 h. Viability was assessed by the MTT assay. CompuSyn software was used to evaluate synergy using the median-effect model.

### Histology

Histological analysis was performed on snap frozen tissue. Briefly, snap-frozen tissue sections fixed in acetone, blocked with 10% FCS and stained with anti-active Caspase 3 (BD Biosciences), cleaved Caspase 8 (Cell Signaling). For p-S6 (Cell Signaling) staining, snap-frozen tissue sections were fixed in 10% neutral buffered formalin and blocked with 5% FCS/ 0.3% Triton X-100 in PBS. Images were taken with an Axiocam 503 color microscope (ZEISS) and quantified using Image J. For conventional immunohistochemistry tumor slides, IHC profiler Image J plugin was used as previously described in detail [32].

### Serum biochemistry

Aspartate aminotransferase (AST), alanine aminotransferase (ALT) and L-Lactatdehydrogenase (LDH) were measured using the automated biochemical analyser Spotchem EZ SP-4430 (Arkray, Amstelveen, Netherlands) and the Spotchem EZ Reagent Strips Liver-1.

### Quantitative RT-PCR

RNA was isolated using Trizol (Invitrogen) and RT-PCR analyses were performed using the iTaq™ Universal SYBR® GreenOne-Step RT-qPCR Kit (Biorad) according to the manufacturer’s instructions. For analysis, expression levels were normalized to *GADPH.*

### Intracellular CAMP assay

Intracellular CAMP levels were determined as per manfacturer’s instructions (Enzo Life Biosciences).

### Mice and in vivo treatments

C57BL/6 J mice were maintained under specific pathogen-free conditions. Seven to nine week old C57BL/6 J mice were subcutaneously injected with 5 × 10^5^ B16F10 cells. Seven days post injection, when tumor volume reached approximately 50 mm^3^, mice were randomized and treated daily for 5 consecutive days with 5 mg/kg Tegaserod or vehicle control (2.5% DMSO in PBS). Tegaserod and vehicle were administered intraperitoneally (i.p.). Tumors were measured using calipers and tumor volume was calculated using the following formula: (tumor length x width^2^)/2. For metastases quantification experiments, C57BL/6 J mice were intravenously injected with 2 × 10^5^ B16F10 cells and treatment with Tegaserod and vehicle (administered i.p.) occurred 1 day post inoculation and continued three times weekly till day 14 post inoculation at which time mice were sacrificed. Metastases from lungs, stored in PBS for short term storage, were manually counted. For survival experiments, C57BL/6 J mice were intravenously injected with 10^5^ B16F10 cells. Treatment with Tegaserod and vehicle (administered i.p.) occurred 1 day post inoculation and continued three times weekly till day 17 post inoculation. Experiments were performed under the authorization of LANUV in accordance with German law for animal protection.

### Data mining using the CCLE

RNA-Seq expression data (Read Count) from the Cancer Cell Line Encyclopedia (CCLE) [16] (Broad Institute and Genomics Institute of the Novartis Research Foundation) for the selected human melanoma cell lines was analyzed using Xena Functional Genomics Explorer [61] and visualized using the MORPHEUS matrix visualization software (https://software.broadinstitute.org/morpheus).

### Statistical analyses

Data are expressed as mean ± S.E.M. Statistically significant differences between two groups were determined using the student’s t-test and between three or more groups, the one-way ANOVA was used with a post-hoc Dunnett test. To assess significance between Kaplan Meier survival curves, the log-rank test was used. Values of *P* < 0.05 were considered statistically significant.

## Supplementary information

**Additional file 1: Figure S1.** B16F10 cells were treated (10 μM-78 nM) for 72 h and IC50 compound values determined. Compounds for which IC50 values were not determinable or were above 10 μM were placed on the 10 μM line. **Figure S2.** (A) Treatment at the indicated time points with TM (3 μM for B16F10 and A375, 5 μM for RPMI and SH4) did not induce changes in CAMP levels (n = 3-4). (B) Treatment with TM for 18 h did not induce changes in serotonin responsive genes. Expression was normalized to GAPDH (n = 3–4). **Figure S3.** TM did not affect the MAPK pathway. Melanoma cells were treated as indicated with increasing concentrations of TM (representative immunoblots of n = 3-5 are shown). Blots are quantified in (B). **Figure S4.** (A) Changes in phospho-S6 (p-S6) at Ser235/6 in MeWo and MEL-JUSO melanoma cells (n = 5–6) following TM treatment are shown. (B) Changes in p-S6 (Ser240/244) (n = 2–3) and p-PDK1 (Ser241) (n = 3–4) following treatment with TM are shown. **Figure S5.** Dose response curves following treatment with the PI3K inhibitor ZSTK474, pan-Akt inhibitor MK-2206 and mTORC1/mTORC2 inhibitor KU-0063794 are shown (n = 3–4). **Figure S6.** (A) TM-treated mice as described in Fig. 4a. experienced a small decrease in body weight (n = 6–8). (B) Treatment with TM did not alter liver damage parameters (n = 3). (C) Quantification of the active Caspase-3 staining for Fig. 4d is shown (n = 6 mice). (D) Immunohistochemical staining of tumor tissue for cleaved Caspase-8 is shown and quantified (n = 6 mice). (E) Tumor lysates probed for cleaved Caspase-8 are shown (n = 6–9 mice, with 3 mice shown). Error bars in all experiments indicate SEM. *P < 0.05 as determined by a Student’s t-test (unpaired, 2 tailed) or a one-way ANOVA with a Dunnett’s post-hoc test.

## Abbreviations

ALT: Alanine aminotransferase
AST: Aspartate aminotransferase
CCLE: Cancer Cell Line Encyclopedia
CREB: CAMP response element binding protein
IC50: Half maximal inhibitory concentrations
JNK: c-Jun N-terminal kinases
LDH: Lactate dehydrogenase
MAPK: Mitogen-activated protein kinase
NCC: NIH Clinical Collection
PKA: Protein kinase A
TM: Tegaserod
TUNEL: Terminal deoxynucleotidyl transferase dUTP nick end labeling
