# Supplementary material for: Repurposing the serotonin agonist Tegaserod as an anticancer agent in melanoma: molecular mechanisms and clinical implications
Source: J Exp Clin Cancer Res. 2020 Feb 21;39:38. doi: 10.1186/s13046-020-1539-7 (PMC7035645; doi:10.1186/s13046-020-1539-7)

# Supplementary Figure 1

**A**

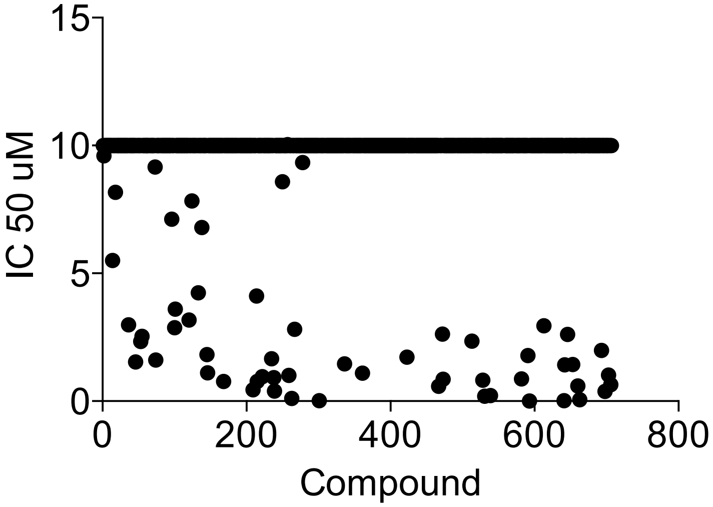

# Supplementary Figure 2

**A**

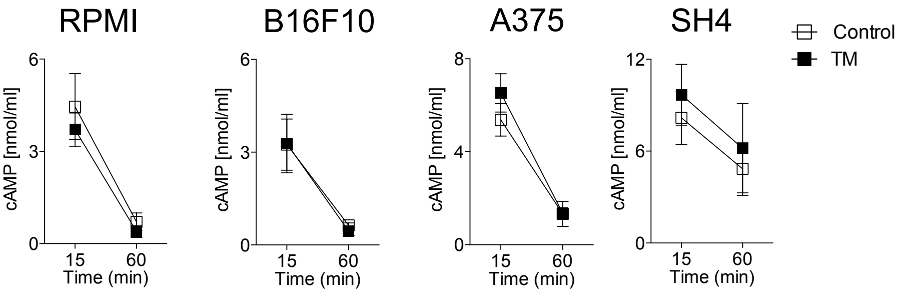


**B**

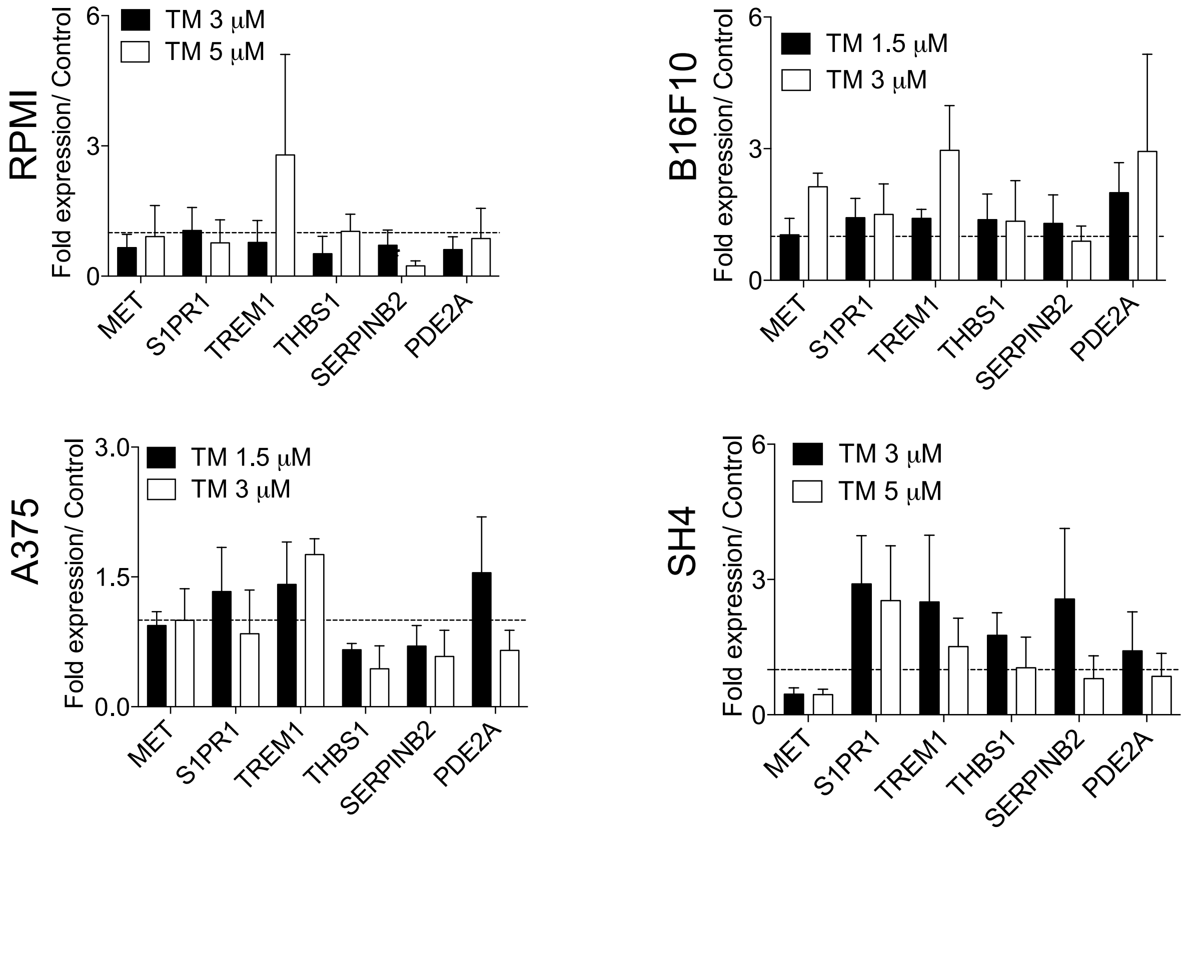

# Supplementary Figure 3

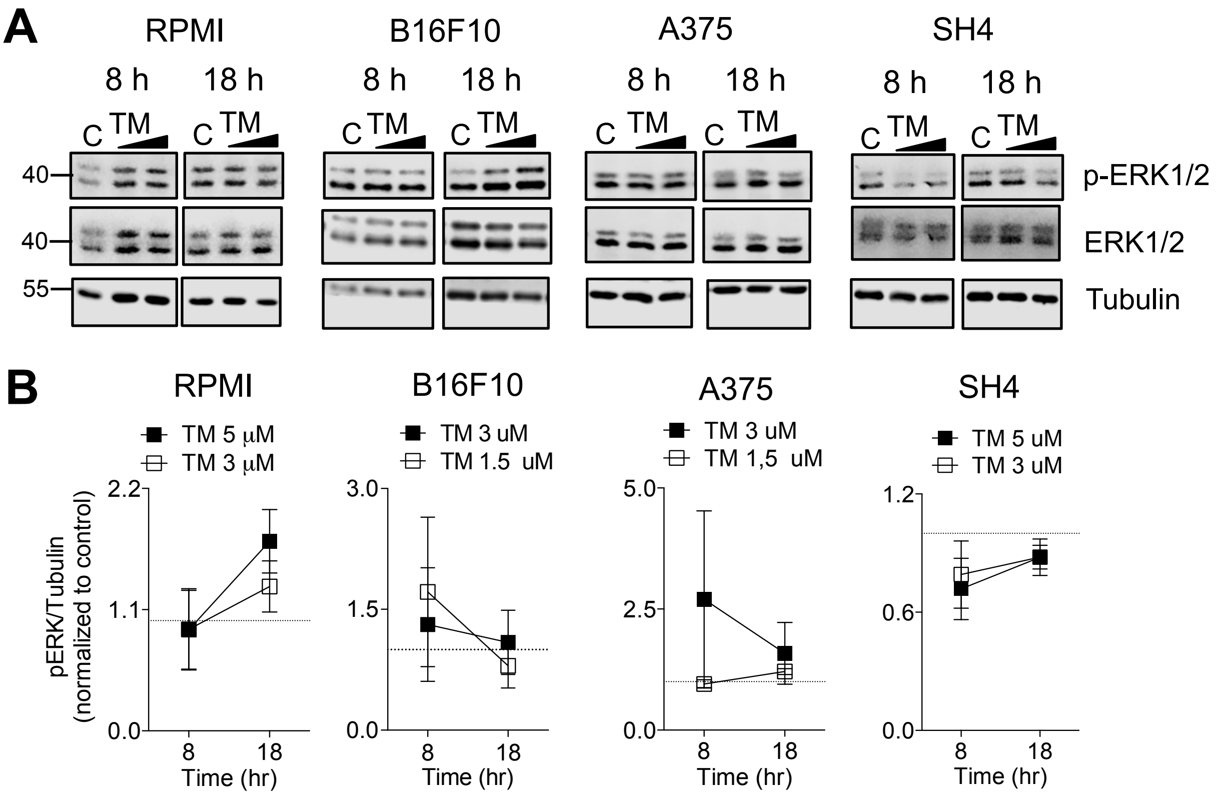

# Supplementary Figure 4

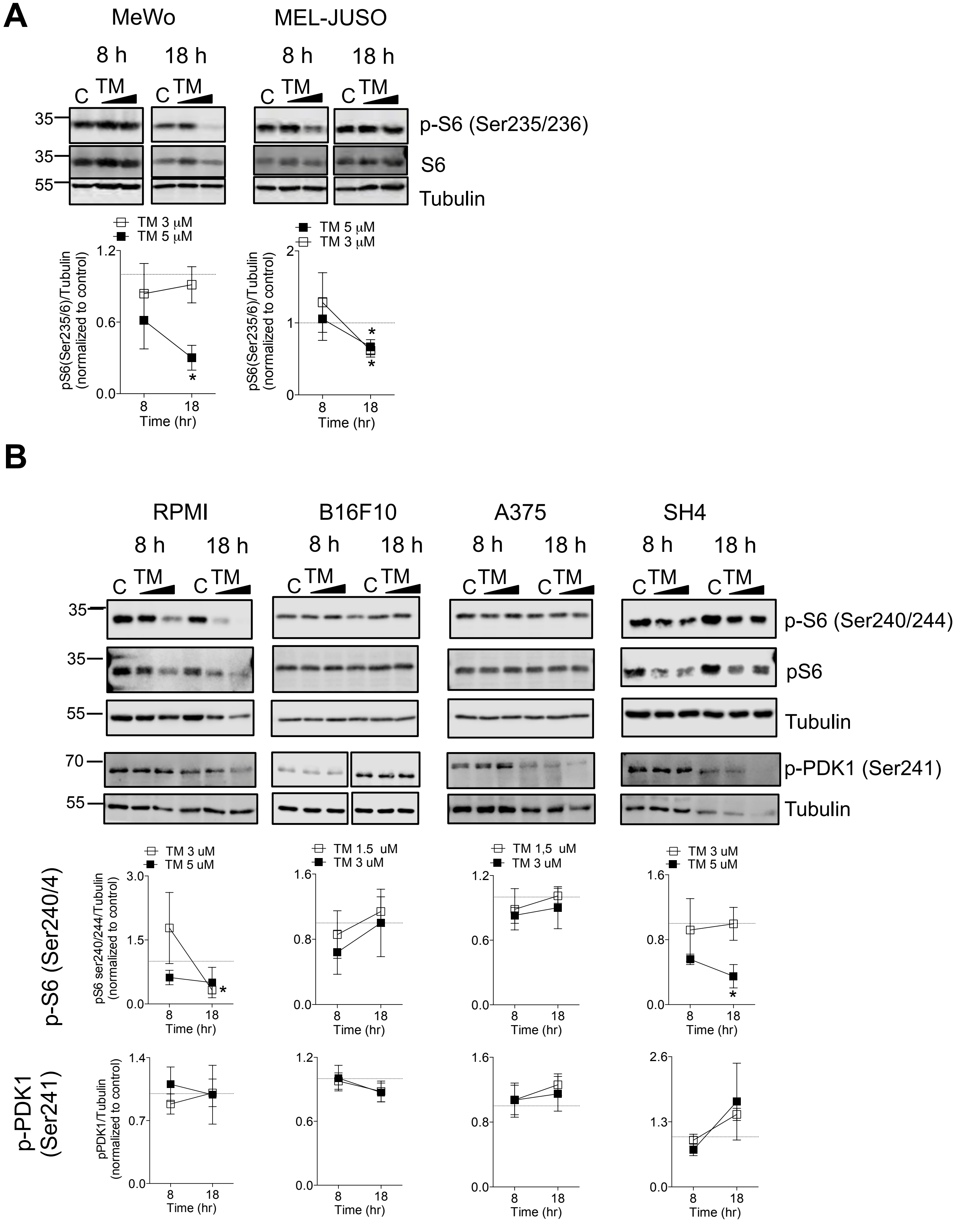

# Supplementary Figure 5

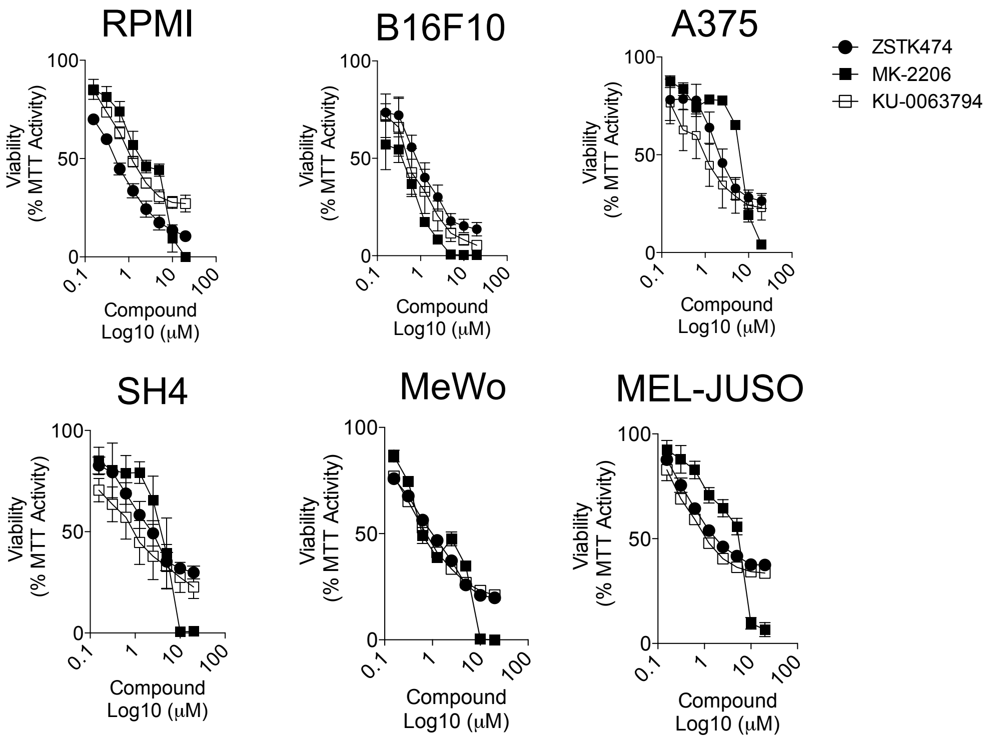

# Supplementary Figure 6

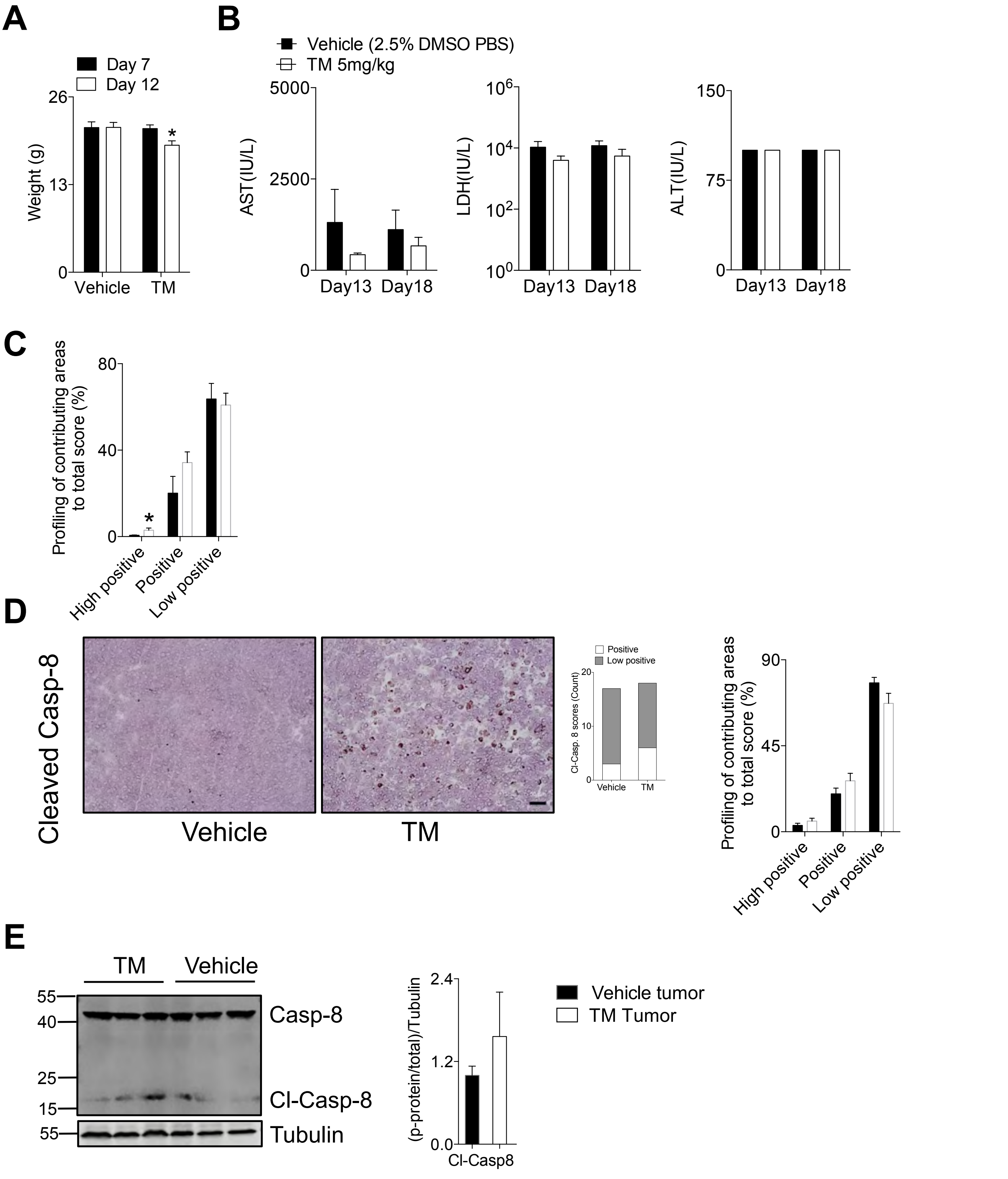

Supplement: Supplementary file 1 — Additional file 1: Figure S1. B16F10 cells were treated (10 μM-78 nM) for 72 h and IC50 compound values determined. Compounds for which IC50 values were not determinable or were above 10 μM were placed on the 10 μM line. Figure S2. (A) Treatment at the indicated time points with TM (3 μM for B16F10 and A375, 5 μM for RPMI and SH4) did not induce changes in CAMP levels (n = 3-4). (B) Treatment with TM for 18 h did not induce changes in serotonin responsive genes. Expression was normalized to GAPDH (n = 3–4). Figure S3. TM did not affect the MAPK pathway. Melanoma cells were treated as indicated with increasing concentrations of TM (representative immunoblots of n = 3-5 are shown). Blots are quantified in (B). Figure S4. (A) Changes in phospho-S6 (p-S6) at Ser235/6 in MeWo and MEL-JUSO melanoma cells (n = 5–6) following TM treatment are shown. (B) Changes in p-S6 (Ser240/244) (n = 2–3) and p-PDK1 (Ser241) (n = 3–4) following treatment with TM are shown. Figure S5. Dose response curves following treatment with the PI3K inhibitor ZSTK474, pan-Akt inhibitor MK-2206 and mTORC1/mTORC2 inhibitor KU-0063794 are shown (n = 3–4). Figure S6. (A) TM-treated mice as described in Fig. 4a. experienced a small decrease in body weight (n = 6–8). (B) Treatment with TM did not alter liver damage parameters (n = 3). (C) Quantification of the active Caspase-3 staining for Fig. 4d is shown (n = 6 mice). (D) Immunohistochemical staining of tumor tissue for cleaved Caspase-8 is shown and quantified (n = 6 mice). (E) Tumor lysates probed for cleaved Caspase-8 are shown (n = 6–9 mice, with 3 mice shown). Error bars in all experiments indicate SEM. *P < 0.05 as determined by a Student’s t-test (unpaired, 2 tailed) or a one-way ANOVA with a Dunnett’s post-hoc test. [file 13046_2020_1539_MOESM1_ESM.pdf]
